# The threshold for the McGurk effect in audio-visual noise decreases with development

**DOI:** 10.1038/s41598-018-30798-8

**Published:** 2018-08-17

**Authors:** Rebecca J. Hirst, Jemaine E. Stacey, Lucy Cragg, Paula C. Stacey, Harriet A. Allen

**Affiliations:** 1 University of Nottingham, Nottingham, UK; 2 Nottingham Trent University, Nottingham, UK

## Abstract

Across development, vision increasingly influences audio-visual perception. This is evidenced in illusions such as the McGurk effect, in which a seen mouth movement changes the perceived sound. The current paper assessed the effects of manipulating the clarity of the heard and seen signal upon the McGurk effect in children aged 3–6 (n = 29), 7–9 (n = 32) and 10–12 (n = 29) years, and adults aged 20–35 years (n = 32). Auditory noise increased, and visual blur decreased, the likelihood of vision changing auditory perception. Based upon a proposed developmental shift from auditory to visual dominance we predicted that younger children would be less susceptible to McGurk responses, and that adults would continue to be influenced by vision in higher levels of visual noise and with less auditory noise. Susceptibility to the McGurk effect was higher in adults compared with 3–6-year-olds and 7–9-year-olds but not 10–12-year-olds. Younger children required more auditory noise, and less visual noise, than adults to induce McGurk responses (i.e. adults and older children were more easily influenced by vision). Reduced susceptibility in childhood supports the theory that sensory dominance shifts across development and reaches adult-like levels by 10 years of age.

## Introduction

The ability to combine auditory and visual information (audio-visual integration) in noisy environments is essential in every day life, such as when holding a conversation. Individuals may differ in the extent to which one sense (or modality) alters or is *dominant* over another. The *McGurk effect* exemplifies this, as hearing a voice say “Ba” and seeing a face say “Ga” often results in perception of an alternative syllable such as “Da” or “Tha”^1^. Sensory dominance, or different weighting between sensory modalities results in different perceptual effects. Visual dominance results in a response to the seen mouth movement, auditory dominance a response to the sound and equal weighting results in a fusion of the two. Alternatively, fusion responses have also been interpreted as visual dominance^2^, where the seen mouth movement alters the sound reported by participants.

Modality dominance might be flexible depending on context^3,4^ and age^5–7^. In some audio-visual paradigms, visual influence appears stronger in adults compared with children^5,8,9^. Children also manifest a preference for auditory information when processing multisensory events^5,10–13^. Sloutsky & Napolitano (2003) found that following adaptation to an audio-visual stimulus, adults were more likely to identify a second stimulus as novel if the visual dimension was altered. Conversely, 4-year-olds identified a stimulus as novel if the auditory dimension changed. Furthermore, studies exploring cross-modal interference effects have reported that 4–5-year-olds struggle to inhibit auditory input^14^. Therefore, perception in young childhood appears to be driven by audition.

The perceptual consequences of auditory dominance in childhood are evident in multisensory illusions. Children are more susceptible to illusions in which auditory information modulates vision. In the Sound-Induced Flash Illusion, participants perceive a single flash as two flashes when presented with two concurrent beeps^15^. Innes-Brown *et al*. (2011) found 8–17-year-olds were more susceptible to the Sound-Induced Flash Illusion compared with adults^16^ (however see^17^). Conversely, children appear less susceptible to the McGurk effect, maintaining veridical perception of sound despite incongruent visual information^18,19^. Tremblay *et al*. (2007) found that 5–9-year-olds made correct auditory “Ba” responses on ~60% of incongruent McGurk trials. This dropped to ~20–30% in 10–19-year-olds, suggesting older children are more susceptible to the McGurk effect.

One explanation of developmental differences is that children manifest delayed development of multisensory integration processes^20^ and thus are less susceptible to multisensory illusions. Nevertheless, children experience other multisensory illusions, such as the Sound-Induced Flash Illusion^16^, and susceptibility to the McGurk effect is modulated by sensory weighting in childhood. Children who experienced early visual and hearing impairments are respectively less and more susceptible to the McGurk effect^19,21^. Furthermore, lip reading ability in childhood is correlated with the size of visual contribution in speech perception^22^. These findings provide compelling evidence for a theory of an experienced based shift in sensory dominance, from audition to vision, that may be modulated by the learnt reliability of visual and auditory input.

In line with a role of sensory reliability, the presence of noise in one or both modalities influences sensory dominance and thus, multisensory integration. Everyday environments are inherently noisy, and this influences which sense drives audio-visual integration. During conversation, the listener may utilise both vision and audition to understand speech. However, in noisy environments, visual information may be more informative. If visual information becomes unclear through factors such as impaired sight or poor viewing conditions, then audition may be particularly salient. In line with this, current theory suggests that the brain weights sensory inputs according to their relative reliability to derive the most accurate percept possible^23–26^. Following this, adults are more susceptible to the McGurk effect in auditory noise^27,28^, and less susceptible in visual noise^27,29^ as audition becomes respectively less and more reliable. This also explains why children with early visual and hearing impairments are respectively less and more susceptible to the McGurk effect^19,21^. Thus, sensory dominance can be modulated within an individual by manipulating the reliability of sensory information.

Recent findings show that when *both* vision and audition are degraded, the McGurk illusion persists. Stacey, Howard, Mitra, & Stacey, (2017) degraded visual and auditory information in McGurk stimuli through introducing blur and white-noise respectively. In line with previous findings, the McGurk effect increased in high levels of auditory noise and decreased when visual information was degraded. Interestingly, McGurk perception remained robust even when both visual and auditory information were degraded; participants still perceived the effect on 66% of trials.

Noisy environments have an everyday impact on audio-visual integration and perception at every stage of life. Yet the effect of *combined* visual and auditory noise upon the McGurk effect in children remains unexplored. To our knowledge, no studies have explored the influence of visual noise on the McGurk effect in children, and only one study has examined auditory noise. Sekiyama & Burnham, (2008) tested the McGurk effect in 6-, 8-, 11-year-olds and adults using four levels of auditory noise. Children were less susceptible to the effect, nevertheless auditory noise increased the effect in both children and adults.

Multiple studies have examined the effect of noise on the McGurk effect in adults^27–31^. However, to our knowledge, none have exploited the effect of manipulating stimulus clarity to derive a threshold for the McGurk effect. A psychophysical approach to measuring sensory weighting in the McGurk effect is informed by computational models of McGurk perception. The Noisy Encoding of Disparity (NED) Model proposed by Magnotti and collegues^32^ proposes that individual differences in McGurk perception may be accounted for by differences in sensory disparity, sensory noise and individual “disparity threshold”, a point at which noise in one modality becomes high enough to prevent fused precepts. An implication of this model is that manipulating sensory noise in one or the other modality may provide an indication of individual differences in thresholds for the effect. This has a benefit over previous approaches, which have used group means, as it provides an indication of how weighting between vision and audition may differ between individuals, change with development, and produce differing thresholds for audio-visual illusions.

In the current study we explored the effect of auditory and visual signal quality on McGurk responses across development to derive thresholds for McGurk responses. The threshold was defined as the noise level inducing non-auditory responses 50% of the time – reflecting the point at which vision prevents correct auditory perception.

Specifically, we hypothesised that:

- Adults would show more McGurk responses than children (regardless of noise level).
- The frequency of McGurk responses would increase across development.
- Although auditory and visual noise were expected to increase and decrease the McGurk effect respectively in adults and children, we hypothesised that adults would show a lower threshold for the McGurk effect compared with children (i.e. require more visual noise to abolish the effect and less auditory noise to induce the effect or, in other words, would require less auditory noise to prevent correct auditory, “Ba”, perception and would show visually influenced, non-auditory, responses even with higher visual noise).
- The threshold for the McGurk effect would progressively decrease across childhood.

## Method

### Participants

To accurately judge the required sample size required to detect an effect of noise on the McGurk effect (required for calculating thresholds) an a priori power analysis was conducted in G*power v3.1 to detect a Cohen’s d of 0.8 in a 2 (sensory condition) × 5 (noise level) ANOVA (see Supplementary Material). This effect size was used based on the large effect sizes reported in the literature for the effect of noise on McGurk responses^27,29^. This analysis governed the size of our adult sample (n = 32). The sample size of our child sample was based on opportunity, however we were confident that it would exceed this number (data were gathered at a large public engagement event and all children had the opportunity to participate). Following data collection, the sample size and age distribution within our child sample permitted a separation of children into three age groups, 3–6-year-olds (n = 29), 7–9-year-olds (n = 32) and 10–12-year-olds (n = 29), enabling a more thorough comparison between different stages of childhood and adulthood.

Thirty-two young adults (Mean age 26.66 years; range 20–35 years; 19 female; 31 right handed) were recruited. Participants were staff and students of the University of Nottingham. They reported having normal or corrected to normal vision and hearing and were fluent English speakers (28 reported English first language, 1 Portuguese, 1 Icelandic, 1 Chinese and 1 Catalan).

Ninety-six children (Mean age 8.1 years; range 3.92–12 years; 47 female) were recruited via Summer Scientist Week (www.summerscientist.org), a public engagement event at the University of Nottingham. Following data collection, children were grouped into three evenly distributed groups for analysis; 3–6-year-olds, 7–9-year-olds and 10–12-year-olds. All participants were fluent English speakers (84 reported that English was the primary language used at home, 4 reported that English was used at home alongside a second language and 8 reported that another language was used at home (1 Russian,1 Japanese, 1 Chinese, 1 Portugese, 2 Telugu, 2 Tamil). Children were rewarded for their time with tokens to take part in other activities. Four 3–6-year-olds were excluded because they did not complete the task. Two 7–9-year-olds were also excluded as parents reported sensory processing difficulties (a perforated ear-drum and sensory processing disorder). Thus, a final sample of 90 children was available for analysis; twenty nine 3–6-year-olds (14 female, Mean age 5.6 years; range 3.92–6.92 years), thirty two 7–9-year-olds (18 female, Mean age 8.3 years; range 7–9.75 years), twenty nine 10–12-year-olds (14 female, Mean age 10.97 years; range 10–12.08 years) and 32 adults.

A large subset of the sample of children also completed measures of language ability/vocabulary knowledge (British Picture Vocabulary Scale: BPVS^33^) and social aptitude (Social Aptitude Scale^34^; n = 82 and n = 78 respectively). Additional exploratory analyses of these data and their relation to McGurk responses are available within the Supplementary Material.

### Equipment

Visual stimuli were presented via a Macbook Air on a Lenovo LT2423 24″ LED Backlit LCD monitor (resolution 1920 × 1080 @ 60 Hz) presented at a viewing distance of ~57 cm. Auditory stimuli were presented via Senheiser eH150 headphones. A Targus numerical response pad was used to gather responses.

### Stimuli

**Figure 1:**
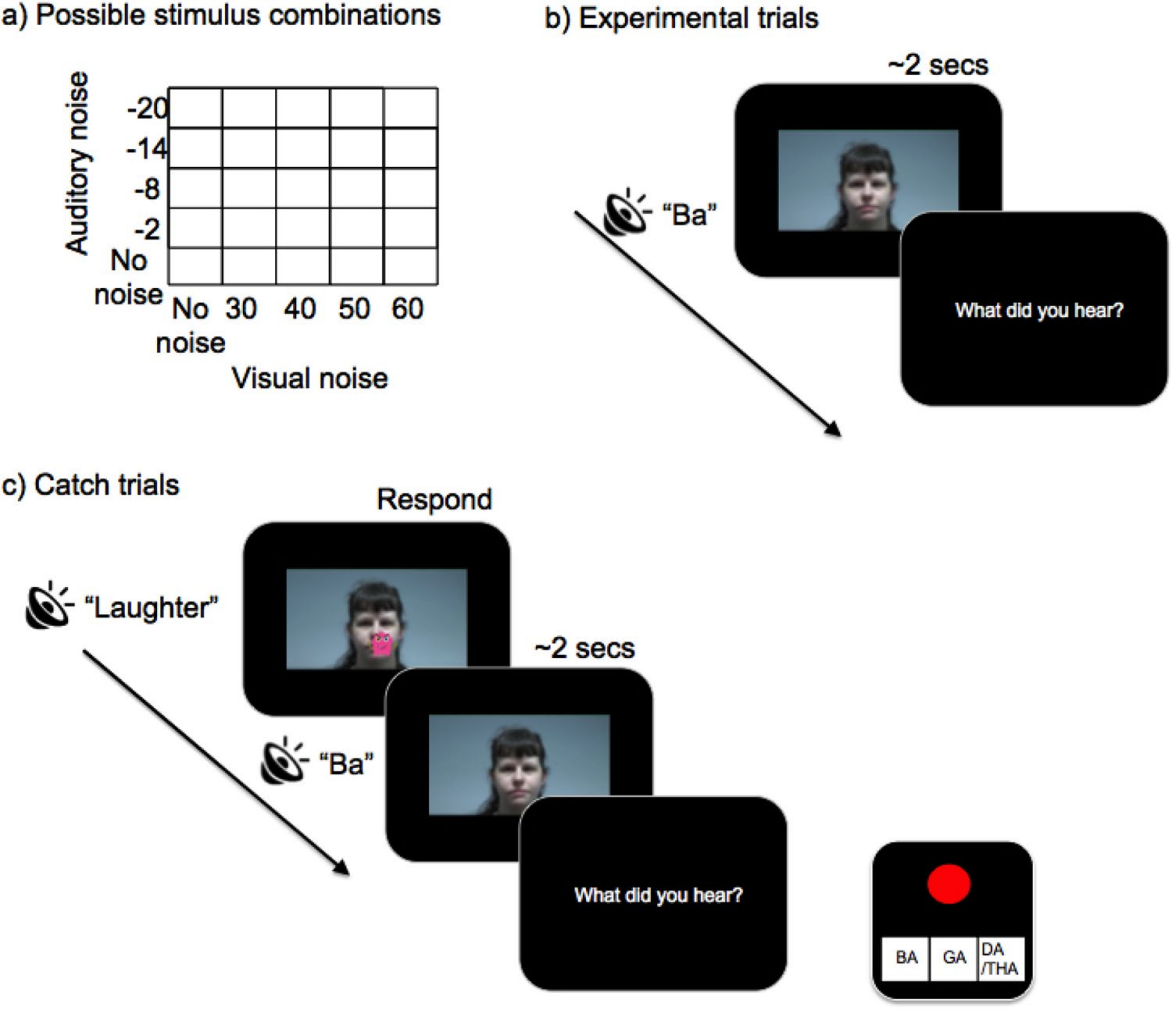
(**a**) Possible stimulus combinations given 5 levels of auditory noise (SNR) and 5 levels of visual noise (% blur). There were 25 congruent “Ba”, 25 congruent “Da”, 25 congruent “Ga” and 25 incongruent auditory “Ba” visual “Ga” trials. (**b**) Experimental trials: Participants watched a 2 second video accompanied with either a congruent or incongruent sound. When asked “what did you hear?” they responded using one of three buttons. (**c**) Catch trials: The video froze at the beginning and the monster cartoon/laughter sound effect was presented. Participants pressed the red button as fast as possible and then the trial continued as normal.

Stimuli were created by splicing together auditory and visual components using Adobe Premiere Pro. Stimuli consisted of videos of a single female speaker vocalising one of three syllables; “Ba”, “Ga” or “Da”. On congruent trials (75% of trials) congruent auditory stimuli were presented (25% “Ba”, 25% “Ga”, 25% “Da”). On incongruent trials (25% of trials), a visual “Ga” and an auditory “Ba” were presented. The proportion of incongruent trials used were comparable, if slightly higher, than those used in previous studies^18,19,21^. The same female speaker was used for all test trials and two different female speakers were used for the practice trials. Videos displayed the head and shoulders of the speaker against a plain white background (size 40 × 21 cm, 2 seconds duration, audio = 41000 Hz, 16 bit). Five levels of visual noise were created via Premiere Pro using the Gaussian blur function (0%, 30%, 40%, 50% and 60% blur). For the purposes of this manuscript both auditory noise and visual blur are referred to as noise, although blurring is a reduction in quality of the signal rather than strictly added ‘noise’. Syllables were presented either without noise or alongside white noise at 4 Signal-to-Noise-Ratios (SNRs; −2 dB, −8 dB, −14 dB and −20 dB). All stimuli were presented at the same sound level (50 dB) determined using an artificial ear (Brüel & Kjær Type 4153). This intensity was clearly audible for all participants as accuracy for syllables in the absence of noise was persistently high (>80% see Supplementary Material). The five levels of auditory and visual noise were combined to produce 25 levels of stimulus quality per syllable (see Figure 1a). There were therefore 100 trials, 25 stimuli per condition (one per each possible noise level). On 10% of trials (see below) a pink cartoon monster (4 × 3.5 cm) appeared covering the mouth, alongside a laughter sound effect. One of these “catch trials” was presented randomly within each 10 trial block.

### Procedure

Adult participants completed the task in a quiet testing lab at the University. Child participants completed the task in a quiet room at the University alongside other studies taking place.

Within each trial, a video was presented followed by an on-screen message asking “What did you hear?” (Fig. 1b) after which participants could respond using three counterbalanced response keys (“Ba”, “Ga” or “Da”/“Tha” – Fig. 1). “Da” and “Tha” were mapped to the same response option in line with previous literature^30,35^. If children could not read the labels they were asked to vocalise their responses and the experimenter would press the button. Once a response had been made the next trial began immediately. Previous research with children has used up to 6 response options^19^ or allowed an open-ended vocalised response^18,21^, thus, three response keys were judged to be appropriate.

Participants first completed five, randomly selected, congruent practice trials in which the spoken syllable was presented in the absence of any noise. Practice trials were followed by 10 blocks of 10 trials in a randomised order. Following each block participants clicked on one of ten treasure chests on the screen, revealing a clue to where a reward token was hidden.

Participants were instructed to focus on the mouth at all times. To ensure attention was maintained upon the mouth, a cartoon monster appeared in the mouth region once per block (See Figure 1c). When the participant saw the monster they pressed a red button, on the same response pad. The trial would not move on until the participant had pressed the red button. All participants included in the analyses successfully completed all 100 trials.

## Analysis and Results

First, we provide a summary of responses to congruent trials. Then we focus on responses to incongruent trials. Following this, we present a threshold analysis to identify the 50% threshold for the McGurk effect (i.e. the point at which McGurk responses were made 50% of the time) in auditory noise (collapsed across visual noise conditions), visual noise (collapsed across auditory noise) and combined audio-visual noise.

### Responses to congruent trials

Accuracy on congruent trials was higher for congruent “Ba” and “Da” stimuli compared with “Ga” stimuli (Table 1). Across groups, participants frequently made “Da/Tha” errors in response to congruent “Ga” stimuli. Increasing visual and auditory noise also lowered accuracy for congruent trials. Interestingly, the effect of auditory, but not visual noise, interacted with age group. Younger children were less accurate than older children and adults only when there was no auditory noise or the highest levels of auditory noise. Extended analyses of congruent stimuli are provided in the Supplementary Material.

**Table 1:** Mean percentage of each response made to each stimulus (collapsed across all noise conditions) in each age groups.

Stimulus	Visual Ba /Auditory Ba			Visual Ga/Auditory Ga			Visual Da/Auditory Da			Visual Ga /Auditory Ba		
Response	“Ba”	“Ga”	“Da”/“Tha”	“Ba”	“Ga”	“Da”/“Tha”	“Ba”	“Ga”	“Da”/“Tha”	Aud “Ba”	Vis “Ga”	Fus “Da”/“Tha”
3–6	75.45 (2.59)	7.45 (1.22)	17.1 (2.11)	22.21 (1.78)	34.9 (3.42)	42.9 (3.49)	14.07 (1.4)	10.07 (1.3)	75.86 (1.96)	52.96 (2.38)	15.59 (2.19)	31.49 (3.04)
7–9	84.5 (2.47)	5.5 (1.16)	10 (2.01)	17 (1.7)	42.75 (3.26)	40.25 (3.32)	9.125 (1.34)	8.88 (1.24)	82 (1.87)	48.75 (2.27)	20.5 (2.09)	30.75 (2.9)
10–12	84.69 (2.59)	4.276 (1.22)	11.03 (2.11)	10.62 (1.78)	39.45 (3.42)	49.93 (3.49)	6.76 (1.4)	6.21 (1.3)	87.03 (1.96)	45.1 (2.38)	17.93 (2.19)	36.97 (3.04)
Adults	89 (2.47)	2.625 (1.16)	7.75 (2.01)	5.25 (1.7)	42.25 (3.26	52.13 (3.32)	4.13 (1.34)	4.25 (1.24)	91.63 (1.87)	37 (2.27)	19.88 (2.09)	42.88 (2.9)

Standard error of the mean shown in brackets. Far right columns represent responses made on incongruent trials. Aud = Auditory, Vis = Visual, Fus = Fusion.

### Development of the McGurk effect

Throughout our analyses of incongruent trials we use four definitions to consider separate effects; “Visual”, “Auditory”, “Fusion” and “McGurk” responses, defined as follows:

- *Visual responses* - “Ga” response to incongruent McGurk stimuli; reflecting a response to the visually presented information.
- *Auditory responses* - “Ba” response to incongruent McGurk stimuli; reflecting a response to the auditory information.
- *Fusion* responses - “Da”/“Tha” response to incongruent McGurk stimuli - participants fuse auditory and visual information to report a syllable different from both the visual (“Ga”) and auditory (“Ba”; McGurk & McDonald, 1976).
- *McGurk responses* - both visual (“Ga”) and fusion (“Da”/“Tha”) responses; reflecting the point at which visual information influences or prevents veridical perception of auditory information.

Incongruent trials were first analysed by assessing mean visual, auditory and fusion responses, regardless of noise level, between age groups. Note that the proportions of these responses are not independent, since participants can make any of these responses to an incongruent trial.

#### Do adults make more McGurk responses than children?

A 4 (age group: 3–6-year-olds, 7–9-year-olds, 10–12-year-olds and adults) × 3 (response type: “Ba”, “Ga”, “Da/Tha”) ANOVA was used to compare responses made on incongruent trials (Table 1). This showed a main effect of response type (*F(*1.7, 201.29) = 81.861, *p* < 0.001, *η*^2^ = 0.38), participants made more “Ba” responses (M = 45.96%, SE = 1.16) compared with “Da”/“Tha” (M = 18.47%, SE = 1.07) and “Ga” (M = 35.51%, SE = 1.48), and more “Ga” than “Da”/“Tha” responses (*p* < 0.001 for all comparisons).

**Figure 2:**
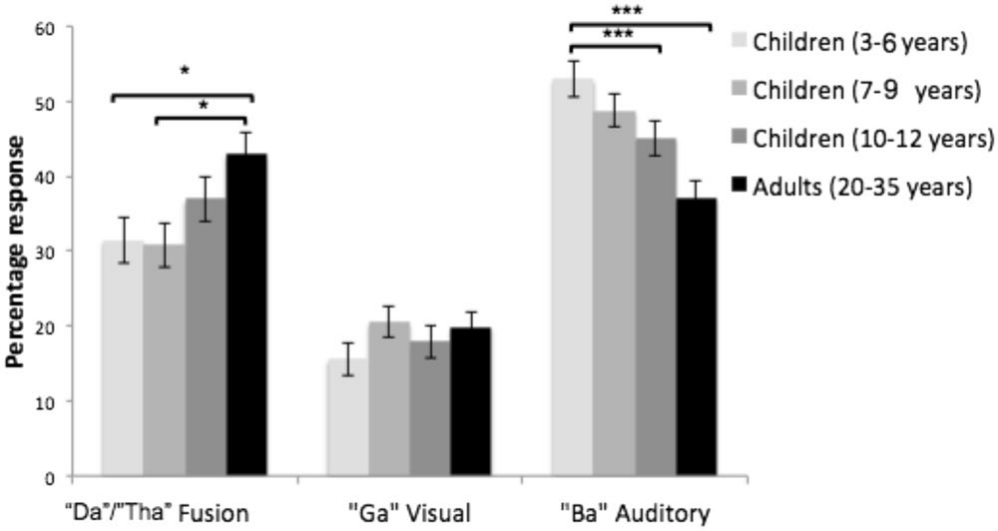
Mean number of “Da”/“Tha”, “Ga” and “Ba” responses made on incongruent trials (visual “Ga” paired with auditory “Ba”) in 3–6-year-olds, 7–9-year-olds, 10–12-year-olds and adults. “Da”/“Tha” responses indicate a fusion response (i.e. a response different from either the visual or auditory stimulus presented), “Ga” responses indicate a response to the visual stimulus and “Ba” responses indicate a response to the auditory stimulus. Error bars indicate standard error of the mean.

There was no main effect of age group (*F(*1, 3) = 1.93, *p = *0.128, *η*^2^ = 0.05), but an interaction between response type and age group (*F(*15.12, 201.29) = 4.49, *p* = 0.001, *η*^2^ = 0.09). This occurred because the effect of age was significant for fusion (“Da”/“Tha”) responses (*F(*3, 118) = 3.73, *p =*0.013, *η*^2^ = 0.09) and auditory (“Ba”) responses (*F(*3, 118) = 8.61, *p <*0.001, *η*^2^ = 0.18) but not visual (“Ga”) responses (*F(*3, 118) = 1.06, *p = *0.368, *η*^2^ = 0.03). As shown in Fig. 2, adults made more fusion responses than 3–6-year-olds (*p *= 0.045) and 7–9-year-olds (*p *= 0.022). However adults did not differ from 10–12-year-olds (*p *= 0.972), and child groups did not significantly differ from one another (all *p* values > 0.849).

Correct auditory “Ba” responses were higher 3–6-year-olds and 7–9-year-olds versus adults (*p* < 0.001 and *p *= 0.002 respectively). However, 3–6-year-olds and 7–9-year-olds did not significantly differ from one another (*p* = 1). 10–12-year-olds, did not significantly differ from adults or other child groups (all *p* values> 0.09).

#### Do McGurk responses increase with development?

**Figure 3:**
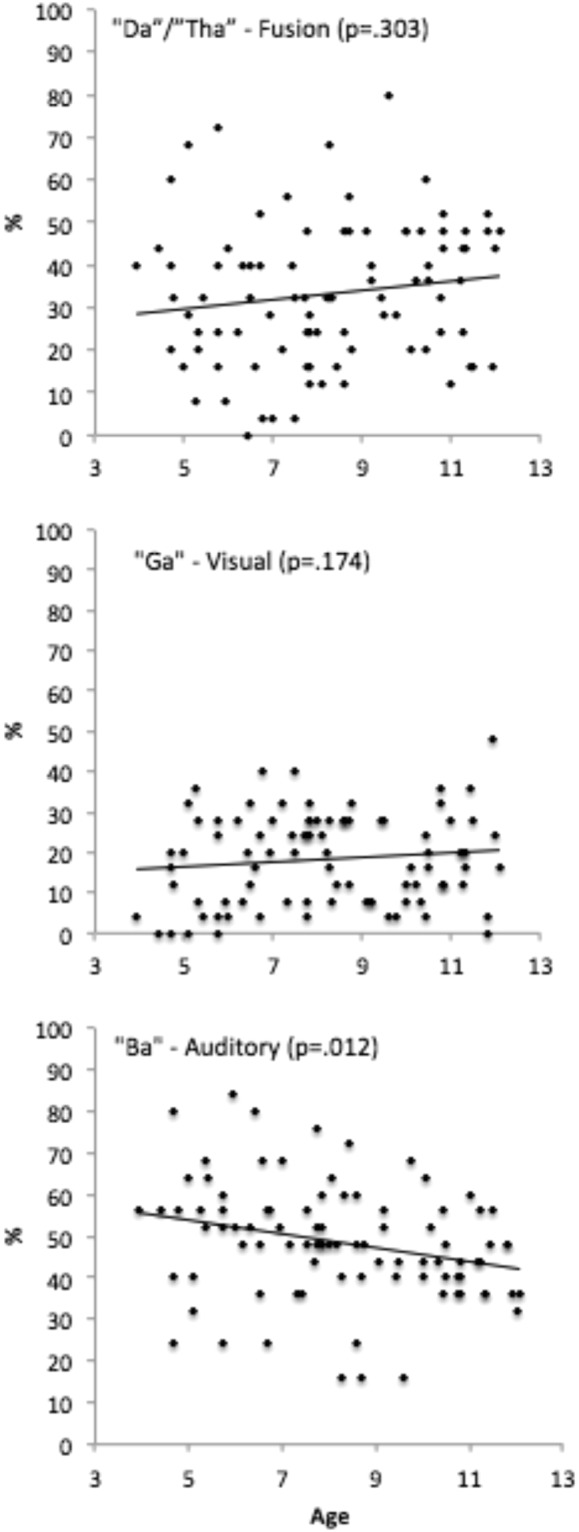
Correlation plots between age and fusion (**a**), visual (**b**), and auditory (**c**) responses to incongruent trials.

To assess whether responses made to incongruent trials could be predicted by age across childhood, three linear regression models were fitted to explore whether the percentage of fusion (“Da/Tha”), visual (“Ga”) and correct auditory (“Ba”) responses on incongruent McGurk trials was predicted by age (Fig. 3). These models found no relationship between age and visual responses (*F*(1, 89) = 1.07, *p *= 0.303, *R *= 0.11, *R*^2^ = 0.01) or fusion responses (*F*(1, 89) = 1.88, *p* = 0.17, *R* = 0.15, *R*^2^ = 0.02), but a negative relationship between age and correct auditory (“Ba”) responses (*F*(1, 89) = 6.64, *p* = 0.012, *R* = 0.27, *R*^2^ = 0.07). Correct auditory responses decreased by 1.57% (SE = 0.06) with every year of age (*t*(89) = 11.81, *p* <0.001).

### Effect of degrading sensory information on McGurk responses in adults and children

Thresholds were defined as the noise level inducing McGurk responses (i.e. “Ga”/“Da/Tha” responses) on 50% of trials, reflecting the point at which vision prevents correct auditory perception.

Three thresholds were identified for each participant for:

1. McGurk responses in auditory noise (collapsed across visual noise conditions; i.e. the y axis of Fig. 1a). Auditory noise would be expected to increase visually-driven responses, so this threshold reflects resistance to visual interference.
2. McGurk responses in visual noise (collapsed across auditory noise conditions; i.e. the x axis of Fig. 1a). Visual noise would be expected to reduce the influence of the visual signal, so this threshold reflects dominance of the visual signal.
3. McGurk responses in combined auditory and visual noise. As only one data point per stimulus level was available for incongruent trials in each participant, a three-dimensional psychometric plane was fitted to data-points, and the threshold was identified as the centroid (mean) coordinate of coordinates yielding 50% accuracy. The change in position of this centroid reflects the audio-visual bias or dominance.

Participants were only included in threshold analyses if their threshold occurred within the range of noise presented. This left twenty two 3–6-year-olds, twenty six 7–9-year-olds, twenty six 10–12-year-olds and twenty one adults available to compare thresholds in auditory noise, nineteen 3–6-year-olds, twenty seven 7–9-year-olds, twenty six 10–12-year-olds and twenty four adults to compare thresholds in visual noise and twenty four 3–6-year-olds, thirty two 7–10-year-olds, twenty nine 10–12-year-olds and thirty two adults to compare thresholds in combined noise. To identify the impact these exclusions had upon the probability of detecting an effect (1−*βerr prob*), post hoc analyses were performed in G*power v 3.1 to assess the likelihood of detecting an effect given a critical alpha of 0.05, the available sample sizes and the observed effect sizes for each comparison^36^. To aid interpretation we report 1−*βerr prob* and *F*_*critical*_ statistics alongside results (probability distribution plots for each analysis are provided in the Supplementary Material). Critically, exclusion of these participants would not have biased our results (i.e. the excluded participants did not show strong effects in the opposing direction to those reported here -see Supplementary Material).

#### The effect of degrading auditory and visual information on McGurk responses in adults and children

**Figure 4:**
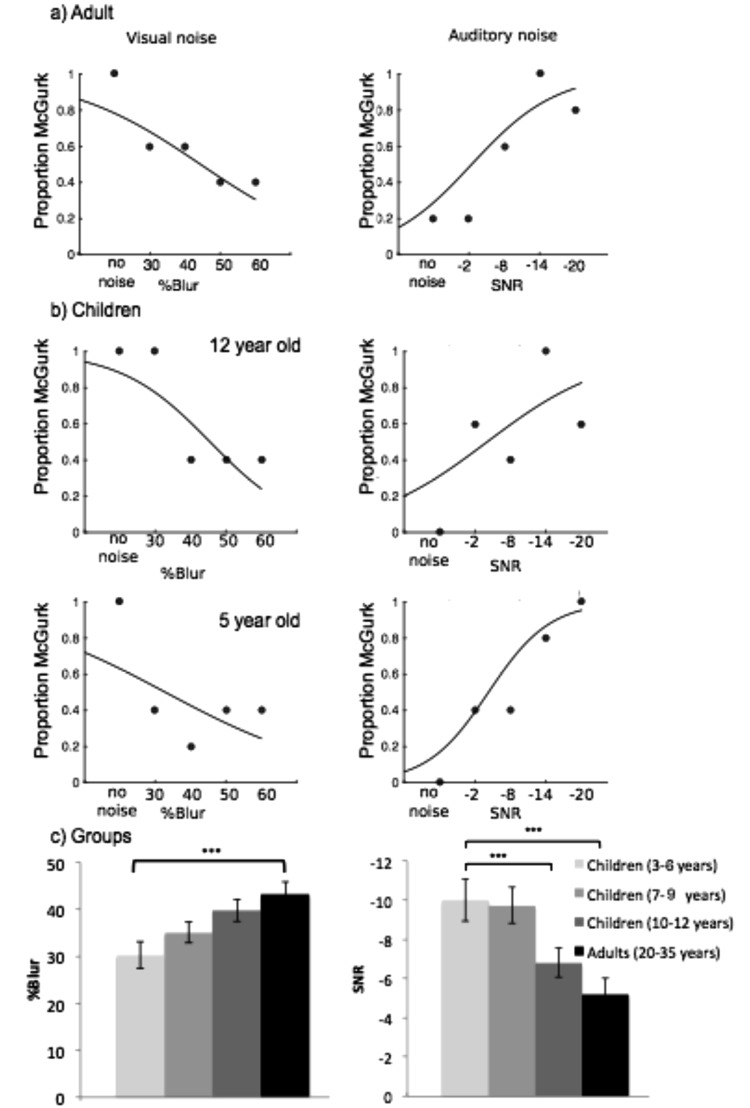
Example psychometric functions showing McGurk responses in (**a**) an adult participant and (**b**) children participants, a 12-year-old and a 5-year-old, and (**c**) average amount of auditory (right) and visual (left) noise required to induce McGurk responses in adults and children (aged 3–6, 7–9 and 10–12 years) with responses collapsed across the opposing noise level. Error bars indicate standard error of the mean.

Table 2 and Fig. 4 show thresholds for McGurk responses in visual and auditory noise *separately*. An ANOVA comparing thresholds for McGurk responses in auditory noise showed a significant effect of age group (*F(*3, 91) = 6.55, *p* <0.001, *η*^2^ = 0.09, 1−*βerr prob* = 0.71, *F*_*critical*_ = 2.7). 3–6-year-olds and 7–9-year-olds did not significantly differ from one another (*p* = 1), both groups required significantly more noise to induce McGurk responses compared with adults (both comparisons *p* =0.003). 10–12-year-olds did not significantly differ from 3–6-year-olds (*p* = 0.076), 7–9-year-olds (*p* = 0.105) or adults (*p* = 1).

**Table 2:** Mean thresholds for McGurk responses in visual and auditory noise (with noise conditions collapsed across the other modality) in each age group.

Age group		Visual Threshold (%Blur)			Auditory Threshold (SNR)		
		*M (SE)*	*Lower CI*	*Upper CI*	*M (SE)*	*Lower CI*	*Upper CI*
Children	3–6 *y*	30.24 (2.83)	24.29	36.18	−10 (1.08)	−12.26	−7.75
	7–9 *y*	35.09 (2.2)	30.57	39.6	−9.72 (0.9)	−11.59	−7.86
	10–12 *y*	39.84 (2.38)	34.94	44.74	−6.8 (0.75)	−8.33	−5.26
Adults		43.15 (2.81)	37.33	48.96	−5.15 (0.84)	−6.91	−3.4

*M* = mean; *SE* = standard error of the mean; *SNR* = signal-to-noise ratio; *y* = years; *CI* = 95% confidence interval.

A separate ANOVA comparing thresholds for McGurk responses in visual noise also showed a significant effect of age group (*F(*3, 92) = 4.48, *p* =0.006, *η*^2^ = 0.06, 1−*βerr prob* = 0.54, *F*_*critical*_ = 2.7). Adults required more visual noise to eliminate McGurk responses compared with 3–6-year-olds (*p* = 0.006), but did not significantly differ from 7–9-year-olds (*p* = 0.137) or 10–12-year-olds (*p* = 1) groups. 10–12-year-olds also did not significantly differ from 7–9-year-olds (*p* = 0.988) or 3–6-year-olds (*p* = 0.072) and 7–9-year-olds did not differ from 3–6-year-olds (*p* = 1).

#### Developmental trajectory analysis

**Figure 5:**
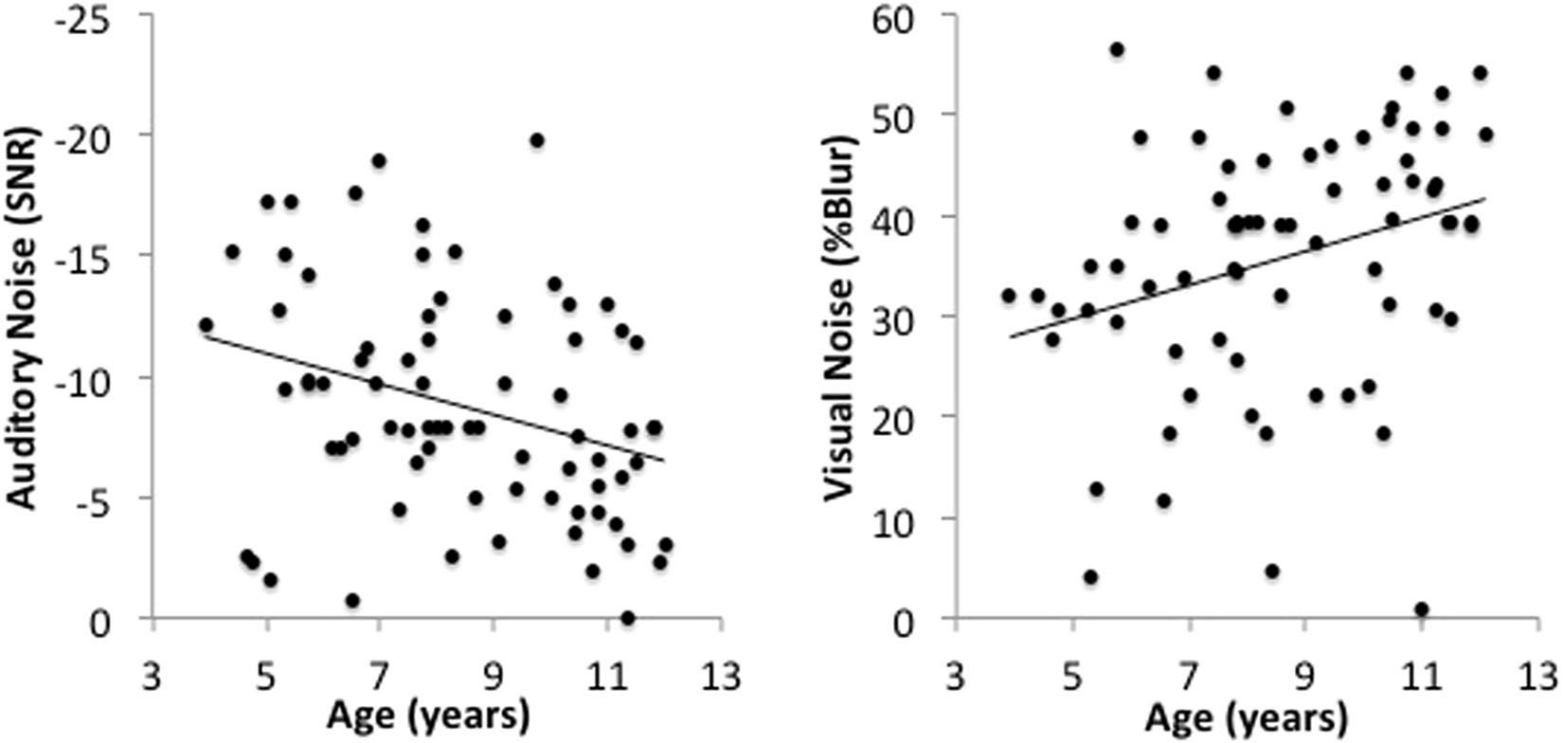
Correlations between age and the amount of auditory noise required to induce McGurk responses (left) and visual noise required to prevent McGurk responses (right) in children. Younger children showed correct auditory responses even in higher levels of auditory noise and lower levels of visual noise compared with older children.

Two linear regression models assessing whether thresholds for McGurk responses could be predicted by age (Fig. 5). These models showed age accounted for a significant proportion of variablity in McGurk responses in auditory noise (*F*(1, 73) = 7.68, *p* = 0.007, R = 0.31, *R*^2^ = 0.10). The auditory noise level required to induce McGurk responses decreased by 0.64 SNR (SE = 0.23) per year (*t*(73) = −7.02, *p* <0.001). Age also accounted for a significant proportion of variablity in McGurk responses in visual noise(*F*(1, 71) = 7.46, *p* = 0.008, R = 0.31, *R*^2^ = 0.10). An increase in 1.74 (% blur, SE = 0.64) was required to eliminate McGurk responses per year (*t*(72) = 3.67, *p* < 0.001).

#### The effect of degrading visual and auditory information

**Figure 6:**
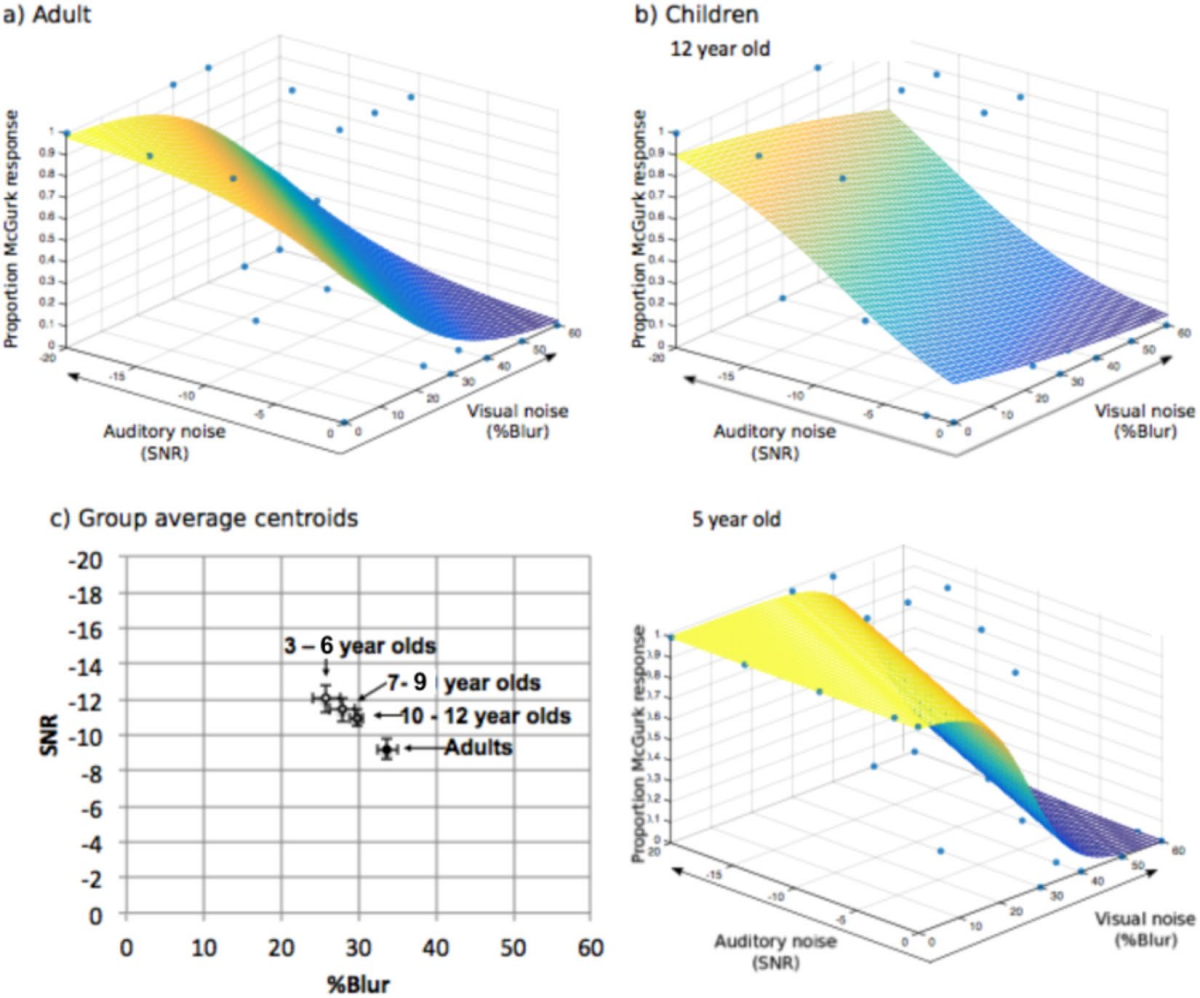
Example psychometric planes fitted for (**a**) a single adult and (**b**) two child participants (aged 12 and 5). The threshold was taken as the centroid co-ordinate of points crossing the 50% threshold for McGurk responses. The average centroid co-ordinates for children and adults are shown in (**c**). Error bars indicate standard error of the mean. There was a significant difference in centroids between groups; adults required more visual noise to prevent McGurk responses and less auditory noise to induce McGurk responses compared with children (i.e. children showed correct auditory responses even in lower levels of visual noise and higher levels of auditory noise compared with adults).

Table 3 and Fig. 6 show thresholds for McGurk responses in *combined* visual and auditory noise. Two ANOVAs compared whether thresholds differed along the auditory and visual noise axes between age groups. Significant effects of age group occurred for the amount of visual noise (*F(*3, 91) = 5.52, *p* =0.001, *η*^2^ = 0.13, 1−*βerr prob* = 0.95, *F*_*critical*_ = 2.69) and auditory noise (*F(*3, 91) = 3.81, *p* = 0.012, *η*^2^ = 0.09, 1−*βerr prob* = 0.81, *F*_*critical*_ = 2.69) required to eliminate and induce McGurk responses.

**Table 3:** Mean threshold positions for McGurk responses in combined visual and auditory noise in each age group.

Age group		Visual Axis (%Blur)			Auditory Axis (SNR)		
		*M (SE)*	*Lower CI*	*Upper CI*	*M (SE)*	*Lower CI*	*Upper CI*
Children	3–6 *y*	25.80 (1.81)	22.06	29.55	−12.05 (0.78)	−13.67	−10.44
	7–9 *y*	27.85 (1.63)	24.52	31.19	−11.42 (0.66)	−12.77	−10.07
	10–12 *y*	29.74 (0.86)	27.98	31.49	−10.95 (0.49)	−11.95	−9.95
Adults		33.72 (1.3)	31.07	36.38	−9.20 (0.59)	−10.4	−8

*M* = mean; *SE* = standard error of the mean; *SNR* = signal-to-noise ratio; *y* = years; *CI* = 95% confidence interval.

In combined noise, adults required significantly more visual noise to prevent McGurk responses compared with 3–6-year-olds (*p* = 0.001) and 7–9-year-olds (*p* = 0.018) but did not significantly differ from 10–12-year-olds (*p* = 0.278). Thresholds did not significantly differ between 10–12-year-olds and 3–6-year-olds (*p* = 0.405), 10–12-year-olds and 7–9-year-olds (*p* = 1) or 7–9-year-olds and 3–6-year-olds (*p* = 1). Adults also required less auditory noise to induce McGurk responses compared with 3–6-year-olds (*p* = 0.014) but did not differ from 7–9-year-olds (*p* = 0.063) or 10–12-year-olds (*p* = 0.284). Thresholds did not significantly differ between any of the child groups (*p* = 1 for all comparisons).

## Discussion

This study was the first to use a threshold approach, inspired by computational models of McGurk perception^32^ and sensory weighting^23–26^, and combine audio-visual noise to examine developmental shifts in susceptibility to the McGurk effect. This approach exploits the effect of degrading signal quality on McGurk responses to gain a precise measure of sensory weighting, whilst also limiting the number of statistical comparisons (i.e. one threshold versus comparison of means at each noise level).

We hypothesised that adults would show more McGurk responses than younger children and that McGurk responses would increase with development. We also predicted that McGurk responses would be influenced by visual and auditory noise in both adults and children^28^, but that thresholds for McGurk responses would decrease (i.e. less auditory noise, more visual noise) through childhood into adulthood.

### McGurk responses increase with development

Our findings support a developmental shift in sensory dominance. Adults made more fusion responses and fewer correct auditory responses compared with 3–6-year-olds and 7–9-year-olds. However, 10–12-year-olds did not significantly differ from adults. Thus, in line with existing literature^18^, our findings show the influence of vision over audition increases across development, reaching adult-like dominance by 10–12 years. An alternative explanation to sensory dominance is that younger children were poor at integrating auditory and visual information^20^. However, our data show that McGurk responses could be induced in young children depending upon the weighting of auditory and visual clarity (discussed below). Thus, we propose a role of sensory weighting (dominance) in influencing McGurk responses across development. Notably, this is not incompatible with an additional role of lower multisensory integration in childhood.

Interestingly, contrary to a theoretical increase in visual dominance, the frequency of visual (“Ga”) responses to incongruent stimuli did not differ between age groups. One explanation of this is that participants erroneously identified “Ga” stimuli as “Da/Tha”, as shown in the analysis of congruent trials. Therefore, some “Da/Tha” responses may have actually reflected (incorrect) visual responses, rather than a fused percept. Nevertheless, increased fusion responses in adults and older children still indicates that visual information was more likely to alter auditory perception in adults compared with younger children.

Notably the range of fusion (“Da”/“Tha”) responses made on incongruent McGurk trials was highly varied in adults (12–76%) and children (0–80%; Fig. 3). Such variance has been reported in adults^35^, the current findings extend this observed variability to childhood. Individual differences in adults have been attributed to variability in fronto-temporal connectivity required for integration^37^. Connectivity differences also likely contribute towards developmental changes, as the underlying neural circuits supporting multisensory integration develop^20,38^. Another possible source of variability in childhood is differing developmental processes such as autistic traits^39^ and language ability^22^. However exploratory analyses (Supplementary Material) found no evidence of a role of either of these factors in altering McGurk responses in our study.

### The threshold for McGurk responses in auditory and visual noise decreases with development

When comparing thresholds for McGurk responses in auditory and visual noise separately, adults required less auditory noise to induce McGurk responses compared with 3–6-year-olds and 7–9-year-olds but not 10–12-year-olds. They also required more visual noise to eliminate McGurk responses compared with 3–6-year-olds, however did not significantly differ from 7–9-year-olds or 10–12-year-olds. Regression analyses also showed threshold shifts occurred progressively across childhood. Thus, the weighting of visual and auditory information (dominance) shifts across development, such that vision influences auditory perception even under higher noise in adults and older children.

Interestingly, when comparing effects in combined noise, adults did not significantly differ from 7–9-year-olds in the auditory noise level inducing McGurk responses, but did significantly differ from 7–9-year-olds as well as 3–6-year-olds in the amount of visual blur required to eliminate McGurk responses. Thus, when auditory and visual signals are both unreliable visual dominance appears immature in 7–9-year-olds (therefore a clearer signal is required for vision to dominate) whilst the influence of audition may be similar to adults (therefore similar auditory noise levels will prevent correct auditory responses). Nevertheless, differences between separate and combined noise comparisons may partially be explained by increased power retained by the combined versus separate comparison (see limitations).

### Visual noise reduces, and auditory noise increases, the McGurk effect in both adults and children

McGurk responses were modulated by stimulus clarity in both adults and children. Increasing visual blur increased the amount of correct auditory responses. Increasing auditory noise decreased the amount of correct responses^30^. Thus, sensory reliability influenced audio-visual integration across age groups.

It might have been expected that children would be more susceptible to auditory noise (given auditory dominance) and therefore require less auditory noise to induce McGurk responses. Conversely, adults might be more susceptible to visual noise (given visual dominance) and require less visual noise to prevent McGurk responses. As this didn’t occur, dominance may map onto an ability to identify a relevant signal (i.e. speech sound or lip movement) within the dominant modality rather than general susceptibility to noise in that modality. This proposal also appears in line with the NED model of McGurk effects, as low sensory disparity (i.e. better detection of signal in noise) in the visual modality relative to the auditory modality this predicts a higher proportion of McGurk responses. This hypothesis is also supported by findings showing children are more sensitive to change in auditory information whilst adults are more sensitive to change in visual information^10^, and findings showing lip reading ability (i.e. detecting a visual signal) predicts a higher influence of vision in speech perception^22^.

### Limitations

We are aware of several limitations in the current study that should be considered. Primarily, our task was limited in the number of trials presented (100 trials 25 of which were incongruent). This limitation was due to the maximum time available for testing each child at Summer Scientist week (15 minutes per child) and was necessary to maintain young children’s attention throughout the task (all children included in analyses completed all 100 trials). The number of trials used was, however, comparable to previous studies assessing the McGurk effect in children^21,40^. Nevertheless, gathering data from more trials over multiple testing sessions would enable fitting of separate two-dimensional psychometric functions to derive thresholds at each level of combined audio-visual noise (through holding noise constant in one modality and varying noise in the other). The findings from the current study provide strong justification for a more in depth investigation of developmental shifts in the McGurk effect using such an approach.

A second limitation to consider is that not all participants could be included in threshold comparisons. This was because derived thresholds fell outside the range of noise presented. We thus report the likelihood of our remaining sample sizes being able to detect effects. These statistics indicated that the primary comparison hindered was the effect of visual noise (collapsed across auditory noise conditions) as the test was limited to a 53% likelihood of rejecting the null. Within this comparison we found a significant difference between the youngest child group and adults, whilst other child groups did not significantly differ from adults. However, given the reduced sensitivity of this test these null findings should be interpreted with caution. Fortunately a more sensitive insight is gained when observing the effect of visual noise in our combined noise comparison, which retained a 94% probability of detecting true effects. Interestingly, in this comparison both 3–6-year-olds and 7–9-year-olds differ from adults in the amount of visual noise required to eliminate McGurk responses.

## Conclusions

We found the threshold for the McGurk effect in audio-visual noise was lower in adults compared with 3–6-year-olds and 7–9-year-olds, but not 10–12-year-olds. Visual noise reduced McGurk responses and auditory noise increased McGurk responses in both adults and children; however the threshold for McGurk responses was lower in adults compared with younger children. These results suggest that susceptibility to the McGurk effect progressively increases, supporting a shift from auditory dominance in childhood towards adult-like visual dominance by the age of 10–12 years.

### Ethical conduct

The methodology included here were approved by the University of Nottingham School of Psychology ethical review board and conducted in accordance with the declaration of Helsinki. Informed consent was obtained from all adult participants in addition to parents/guardians of child participants and assent was obtained from child participants.

## Electronic supplementary material

Supplementary material

## Data Availability

The datasets generated and/or analysed during the current study are available from the corresponding author on reasonable request.
