# Supplementary material for: The threshold for the McGurk effect in audio-visual noise decreases with development
Source: Sci Rep. 2018 Aug 17;8:12372. doi: 10.1038/s41598-018-30798-8 (PMC6098036; doi:10.1038/s41598-018-30798-8)
Supplement: Supplementary file 1 — Supplementary material [file 41598_2018_30798_MOESM1_ESM.docx]

# Supplementary material

# Manuscript: The threshold for the McGurk effect in audio-visual noise decreases with development

Authors: Rebecca, J. Hirst^[[1]](#footnote-1)^*, Jemaine. E. Stacey^[[2]](#footnote-2)^, Lucy Cragg^[[3]](#footnote-3)^1, Paula C. Stacey^[[4]](#footnote-4)^2, Harriet, A. Allen^[[5]](#footnote-5)^1

**Content**

S1 Planned comparison for the effect of noise on McGurk responses in adults

S2 Accuracy in the absence of noise

S3 Summary of results from congruent trials

S4 Details regarding participants excluded from threshold analyses

S5 Post-hoc power distribution plots following participant exclusion

S6 Exploratory analysis: Are social aptitude and vocabulary knowledge related to the McGurk effect in children

# S1. Planned comparison for the effect of noise on McGurk responses in adults

To accurately judge the required sample size required to detect an effect of noise on the McGurk effect (required for calculating thresholds within our main manuscript) an a priori power analysis was conducted in G*power v3.1 based a 2 (sensory condition) x5 (noise level) ANOVA. This indicated a sample size of 32, which governed the size of our adult sample. The sample size of our child sample was based on opportunity, however we were confident that it would exceed this number (data were gathered at a large public engagement event and all children had the opportunity to participate). Within this analysis, as in our paper, McGurk responses are classified as anything that is not a correct auditory response (i.e. “Ga” or “Da”/”Tha”).

To confirm that the effect of noise on the McGurk effect was present in our data a a 2 (sensory condition ) x5 (noise level) ANOVA was carried out. The ANOVA indicated no main effect of sensory condition but a main effect of noise level (*F*(4, 31) = 11, *p* < .001, $\eta^{2}$ = .26) and an interaction between noise level and sensory condition (*F*(4, 124) = 115.325, *p* <.001, $\eta^{2}$ = .79). As indicated in Figure S1, this interaction occurred because increasing visual noise significantly increased the amount of correct auditory responses to incongruent McGurk stimuli. Conversely, increasing auditory noise significantly decreased correct auditory responses to incongruent McGurk stimuli.


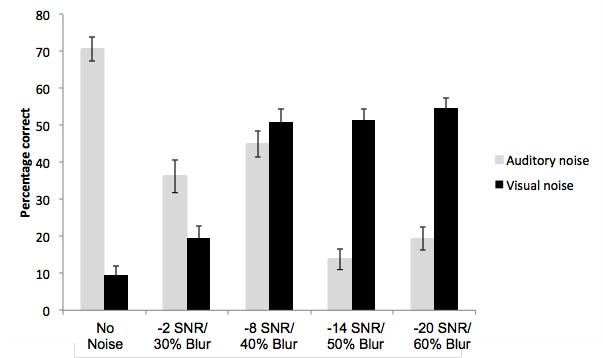


**Figure S1.** Effect of auditory and visual noise on percentage correct auditory responses made to incongruent (auditory “Ba” visual “Ga”) stimuli.

# S2. Accuracy in the absence of noise

To identify how accurate participants were in the absence of noise we analysed accuracy for the 5 practice trials (with two speakers different from that used within the main trials). A 4 (age group: 3-6-year-olds, 7-9-year-olde, 10-12-year-olds and adults) x 3 (syllable: “Ba”, “Ga”, “Da”) showed no main effect of syllable (*F*(1.90, 220.5^[[6]](#footnote-6)^) = .795, *p* = .453, $\eta^{2}$=.01), no main effect of age group (*F*(1, 116) = 2.24, *p* = .087, $\eta^{2}$ = .05) and no interaction between syllable and age group (*F*(5.702, 220.5) = 1.91, *p* = .084, $\eta^{2}$=.05). Mean accuracy for each syllable in each age group are shown in table S1. These high means show that the syllables were detectable for adults and children. Notably, the speaker used within the main trials was different to those presented in practice trials. Nevertheless, when asked to judge congruent “Ba”, “Ga” and “Da” syllables in the absence of any noise within the main trials (for which there was one trial per syllable per participant) accuracy was high (Table S2).

| **Age group** | | **Syllable** | | |
| --- | --- | --- | --- | --- |
|  |  | “Ba” | “Ga” | “Da” |
|  |  | *M* (*SE*) | *M* (*SE*) | *M* (*SE*) |
| Children | 3 – 6 | 89.66 (3.75) | 75.86 (4.76) | 83.91 (4.44) |
|  | 7 – 9 | 91.67 (3.57) | 89.06 (4.53) | 88.54 (4.23) |
|  | 10 - 12 | 88.51 (3.75) | 86.21 (4.76) | 90.81 (4.44) |
| Adults |  | 92.22 (3.69) | 100 (4.68) | 84.44 (4.37) |

**Table S1.** Mean percentage correct response for each congruent syllables in each age group within the practice trials. *M* = mean; *SE* = standard error.

| **Age group** | | **Syllable** | | |
| --- | --- | --- | --- | --- |
|  |  | “Ba” | “Ga” | “Da” |
| Children | 3 - 6 | 96.6% | 79.3% | 100% |
|  | 7 - 9 | 100% | 81.3% | 100% |
|  | 10 - 12 | 100% | 82.8% | 100% |
| Adults |  | 100% | 71.9% | 100% |
| **Table S2.** Percentage of participants in each age group making correct responses to congruent syllables in the absence of noise within the main trials (only one stimulus was presented for each participant. | | | | |

# S3. Summary of results from congruent trials

Within our manuscript we focus on the McGurk effect. This entailed an analysis focused on responses given to incongruent McGurk stimuli – in which auditory information (“Ba”) and visual information (“Da”) conflicted. However, our stimuli also included 25 congruent “Ba”, 25 congruent “Da” and 25 congruent “Ga” stimuli (each presented in 5 levels of auditory noise and 5 levels of visual noise). Here we provide an overview of:

a) Responses made to congruent stimuli (regardless of noise).

b) The effect of auditory noise on accuracy in each of these conditions (regardless of visual noise)

c) The effect of visual noise on accuracy in each of these conditions (regardless of auditory noise).

We expected that increasing auditory and visual noise would reduce accuracy on congruent trials in adults and children.

## S3.1 Overall accuracy for congruent stimuli in adults and children.

A 4 (age group: 3-6-year-olds, 7-9-year-olds, 10-12-year-olds and adults) x 3 (stimulus type: congruent “Ba”, “Ga” and “Da”) x 3 (response type: “Ba”, “Ga”, “Da”/”Tha”) ANOVA showed no main effect of stimulus type (*F(*1.26, 148.42) = 1.6, *p =*.204, $\eta^{2}$ = .01) and no main effect of age group (*F(*3, 118) = 1.93, *p =* .128, $\eta^{2}$ = .05) but a main effect of response type (*F(*1.67, 197.45) = 270.55, *p <* .001, $\eta^{2}$ = .68). This main effect occurred because participants made significantly more “Da”/”Tha” (M = 47.3, SE = .88) compared with “Ga” (M = 17.38, SE = .74; *p* <.001) and “Ba” (M = 35.23, SE = .59; *p* < .001). The proportion of “Ba” responses was also higher than “Ga” (*p* <.001).

There was a significant interaction between response type and age group (*F(*5.02, 197.45) = 2.4, *p =* .029, $\eta^{2}$ = .02). This occurred because whilst there was a main effect of age for “Da”/”Tha” responses (*p* = .027) and “Ba” responses (*p* =.020) there was no effect of age on “Ga” responses (*p* = .565). However, post-hoc pairwise comparisons did not reveal ant differences between age groups in any type of response.

There was also a significant interaction between stimulus type and response type (*F(*2.66, 313.47) = 1110.607, *p <* .001, $\eta^{2}$ = .89). This was because on “Da” trials, the proportion of “Da”/”Tha” responses made were significantly higher than other responses (*p* < .001 for both comparisons) and the proportion of “Ga” and “Ba” responses did not significantly differ from one another (*p* =.609). Similarly, on congruent “Ba” trials the proportion of “Ba” responses was significantly higher than other responses (*p* <.001 for both comparisons) however the proportion of “Da”/”Tha” errors was significantly higher than “Ga” errors (*p* <.001). In contrast to this, participants appeared to confuse congruent “Ga” stimuli with “Da”, the proportion of “Da”/”Tha” and “Ga” responses did not significantly differ from one another (*p* = .148) however both of these responses were made significantly higher than “Ba” responses (*p* <.001) for both comparisons) .

Finally, there was a significant three-way interaction between stimulus type, response type and age group (*F(*7.97, 313.47) = 7.01, *p <=* .001 $\eta^{2}$ = .02). As can be seen in Table 1 of our main manuscript when presented with congruent “Ga” stimuli there was no effect of age group on the type of response made (*p* = .333), and this was likely due to lower accuracy across groups. However, when presented with congruent “Ba” stimuli 3-6-year-olds were significantly more likely to make “Da”/”Tha” (*p* = .01) or “Ga” (*p* =.029) errors compared with adults (although we should emphasise high accuracy rates despite errors). Errors in 3-6-year-olds did not significantly differ from other child groups however, and there was no difference between 7-9-year-olds, 10-12-year-olds and adults. When presented with congruent “Da” stimuli 3-6-year-olds also presented more errors, making significantly fewer correct “Da”/”Tha” responses compared with the 10-12-year-olds (*p* = .001) and adults (*p* <.001). 7-9-year-olds also made significantly fewer correct “Da”/”Tha” responses compared with adults (p = .002), 3-6-year-olds and 7-9-year-olds did not significantly differ from one another in this aspect. Compared with adults, 3-6-year-olds made significantly more “Ga” (*p* = .01) and “Ba” (*p* < .001) errors, and made more “Ba” errors compared with the 10-12-year-olds (*p* =.002).

## S3.2 The effect of auditory noise on accuracy for congruent stimuli in adults and children

A 4 ( age group: 3-6-year-olds, 7-9-year-olds, 10-12-year-olds and adults) x 3 (stimulus type: congruent “Ba”, “Ga” and “Da”) x 5 (auditory noise level: no noise, -2 SNR, -8 SNR, -14 SNR, -20 SNR) ANOVA showed a main effect of stimulus type (*F*(1.70, 200.92) = 371.57, *p* <.001, $\eta^{2}=$.76), a main effect of auditory noise level (*F*(4, 472) = 221.42, *p* <.001, $\eta^{2}=$.64) and a significant effect of age group (*F*(3, 118) = 10.54, *p* <.001, $\eta^{2}=$.21). In line with the above analyses, the main effect of stimulus occurred because accuracy was significantly higher for congruent “Ba” and “Da” stimuli compared with “Ga” (*p* < .001 for both comparisons) and accuracy did not significantly differ between “Ba” and “Da” ( *p* = 1). The main effect of age group occurred because accuracy gradually increased with age (3-6-year-olds: M = 62.07%, SE = 1.6, 7-9-year-olds: M = 69.75%, SE = 1.53, 10-12-year-olds: M = 70.39%, SE = 1.6, adults: M = 74.29%, SE = 1.53). Accuracy was significantly lower in 3-6-year-olds compared with 7-9-year-olds (*p* = .004), 10-12-year-olds (*p* = .002) and adults (*p* < .001). Comparisons between other groups did not reach significance. There was no significant interaction between stimulus type and age group (*F*(5.11, 200.92) = .943, *p* =.465, $\eta^{2}$ = .01).

The main effect of auditory noise occurred because accuracy progressively decreased as auditory noise increased (no noise: M = 91.28%, SE = 1.02; -2 SNR: M = 70.9%, SE =1.15 ; -8 SNR: M = 66.68%, SE = 1.26; -14 SNR: M = 64.66%, SE = 1.31; -20 SNR: M = 52.11%, SE = 1.03). Accuracy was significantly higher with no noise compared to all noise levels (*p* < .001 for all comparisons). Accuracy was also higher at -2 SNR compared with -8 SNR (*p* = .015) and higher levels (*p* < .001 for both). Accuracy did not significantly differ between -8 and -14 SNR (*p* = 1), but at -14 SNR accuracy was higher than – 20 SNR (*p* < .001).

The effect of auditory noise level significantly interacted with both age group (*F*(12, 472) = 2.49, *p* = .004, $\eta^{2}=$ .02) and stimulus type (*F*(6.6, 778.21) = 70.66, *p* < .001, $\eta^{2}=$ .36). The interaction between auditory noise level and age group occurred because age differences were significant in no noise and at the highest two noise levels (*p* = .001 and *p* < .001 respectively), however, age differences did not reach significance at -14 SNR (*p* = .117) and reached marginal significance at -8 SNR (*p* = .045). The interaction between auditory noise level and stimulus type arose due to different effects of noise on the pattern of accuracy for each syllable. As shown in Figure S2, accuracy for “Ba” progressively decreased with each stage of auditory noise but then accuracy increased at the highest noise level. Accuracy for “Ga” significantly decreased from no noise to -2 and -8 SNR, then increased at -14 SNR, then, again, decreased at -20 SNR. For “Da” accuracy progressively decreased at each increment in noise.


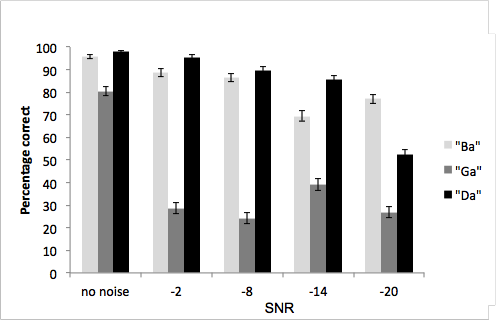


**Figure S2.** Percentage correct (across all age groups) for congruent stimuli at each noise level.

Finally, there was a three-way interaction between auditory noise level, stimulus type and age group (*F*(19.79, 778.21) = 2.5, *p* < .001, $\eta^{2}=$.04). As shown in Figure S3 this occurred because the main effect of age did not reach significance at all levels of noise in each syllable (non significant effects are indicated with dashed boxes). At levels in which the effect of age group reached significance there was a consistent pattern in which accuracy was lower in younger child groups compared with older children and adults.


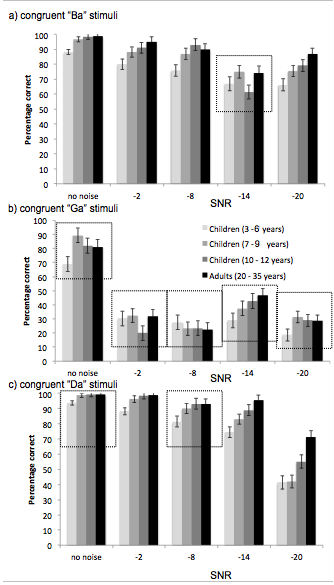


**Figure S3**. Accuracy for congruent stimuli at each auditory noise level in each age group. Dashed boxes indicate levels of noise in each stimulus at which the effect of age was non significant.

## S3.3 The effect of visual noise on accuracy for congruent stimuli in adults and children

A 4 (age group: 3-6-year-olds, 7-9-year-olds, 10-12-year-olds and adults) x 3 (stimulus type: congruent “Ba”, “Ga” and “Da”) x 5 (visual noise level: no noise, 30% blur, -40% blur, 50% blur, 60% blur) ANOVA showed a main effect of stimulus type (*F*(1.701, 200.92) = 371.573, *p* < .001, $\eta^{2}=$ .75), a main effect of visual noise (*F*(4, 472) = 6.882, *p* < .001, $\eta^{2}=$ .05) and a main effect of age group (*F*(3, 118) = 10.54, *p* < .001, $\eta^{2}=$ .21). The main effect of age and stimulus type were identical to those reported above.

The main effect of visual noise occurred because accuracy was significantly higher with no noise compared with 50% (*p* < .001) and 60% blur ( *p* = .003). Accuracy was also higher at 30% blur compared with 50% blur (*p* = .014) – all other comparisons did not reach significance. Notably, there was no interaction between age group and visual noise (*F*(12, 472) = 1.76, *p* = .052, $\eta^{2}=$ .04) suggesting similar effects across age groups.

The effect of visual noise significantly interacted with stimulus type (*F*(8, 944) = 9.672, *p* < .001, $\eta^{2}=$ .07). This interaction occurred because the main effect of visual noise reached significance for “Ba” stimuli (*p* < .001) but not “Ga” (*p* = .264) or “Da” (*p* = .539) stimuli. As can be seen in Figure S4 visual noise gradually reduced accuracy for congruent “Ba” stimuli. There was no significant three-way interaction between age group, stimulus type and visual noise (*F*(20.52, 807.24) = 1.32, *p* = .137, $\eta^{2}=$ .03)


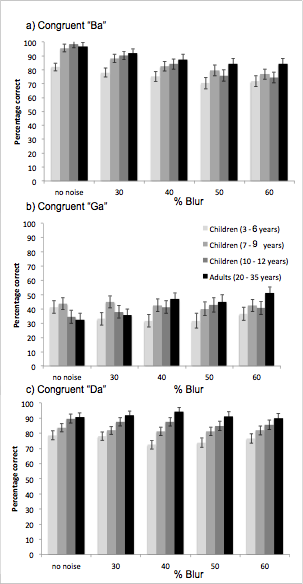


**Figure S4**. Accuracy for congruent stimuli at each visual noise level in each age group.

## S3.4 Discussion

A few points of interest are notable from these supplementary analyses of responses to congruent trials.

1. Accuracy on congruent trials was always lower for “Ga” compared with “Ba” and “Da” trials.
2. Age differences were greatest in higher auditory noise levels.

With regards to point one, accuracy was lower for congruent “Ga” stimuli compared with the other two syllables and participants were equally likely to respond “Da” or “Ga” to these stimuli. This may have meant that for our main analyses of incongruent McGurk stimuli (auditory “Ba” visual “Ga”) participants would be biased towards correct auditory responses. Given this we must be careful in our interpretation of “fusion” versus “visual” responses, as if participants were likely to confuse these stimuli fusion responses may reflect participants responding to what they believe the visual stimulus represents, making it similar to a “visual” response.

With regards to points two, this suggest that children were more influenced by the effect of noise and found it more difficult to identify the correct, congruent, signal in high auditory noise compared with adults. This finding appears contrary to our analysis of McGurk responses, where we saw children’s veridical perception of sound was less influenced by noise compared with adults (who were more easily swayed towards the influence of vision). One explanation of this may be that children struggle to integrate auditory and visual noise in levels of high auditory noise ^1^ and this results in reduced benefit for congruent speech signals. Following this, when auditory and visual signals are in conflict, but also in high levels of auditory noise, reduced integration may result in reduced McGurk perception in children.

# S4. Details regarding participants excluded from threshold analyses

Within our main manuscript, participants were only included in threshold analyses if their estimated threshold occurred within the range of noise presented. This resulted in 75 (of 90) child and 21 (of 32) adult data sets available for the analysis of auditory noise upon McGurk responses and 73 child and 24 adult data sets available for the analysis of visual noise upon the McGurk effect. Due to the large number of exclusions in this analysis, it is important to illustrate that these exclusions did not bias our main analyses.

Figure S5 shows the distribution of estimated thresholds for child (left) and adult (right) participants excluded from analysis of auditory (top) and visual (bottom) analyses. These estimated thresholds must be treated with caution, however, the pattern of results is in line with the effects reported in our main manuscript. Inclusion of these participants would have inflated the effects reported in our paper.

In terms of excluded adult participants adults excluded from analysis of visual thresholds showed thresholds shifts towards increased noise levels, indicating that these participants would have shown McGurk perception even in high levels of visual noise. In terms of auditory noise, excluded adults participants all had thresholds requiring a lower SNR (favouring signal over noise). Thus, these participants would have shown McGurk responses even in lower levels of auditory noise. Critically these data points are in line with our main analyses and removal would not have altered the direction of results.

**Figure S5.** Thresholds of participants who were excluded from analysis of auditory thresholds (upper) and visual thresholds (lower). Participants were excluded as their thresholds fell outside the range of presented intensities (shown with dashed lines).


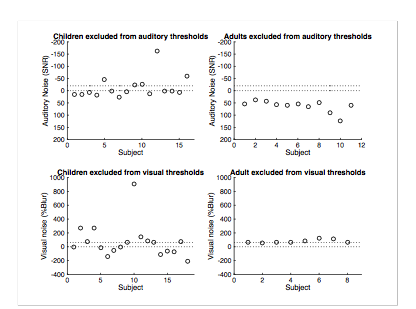


# S5 Post-hoc power distribution plots following participant exclusion

Within our main manuscript we report that some participants had to be excluded from threshold comparisons due to inappropriate thresholds (See also supplementary section S2). We therefore report the likelihood of our sample size being able to detect effects with the remaining sample size ($1-\beta err prob)$. To supplement interpretation of our data, below are the obtained probability distribution plots for obtaining effects with the remaining sample sizes, observed effect sizes and a critical alpha of .05.

**Figure S6.** Probability distributions of accepting the null (H0 – blue line) and the alternative (H1 – red line) hypotheses for each comparison with remaining sample sizes. Shaded areas show probability of Type II (blue) and Type I (red) errors (plots produced using G*power v3.1). Actual F value obtained is displayed relative to the critical F with the dashed line.


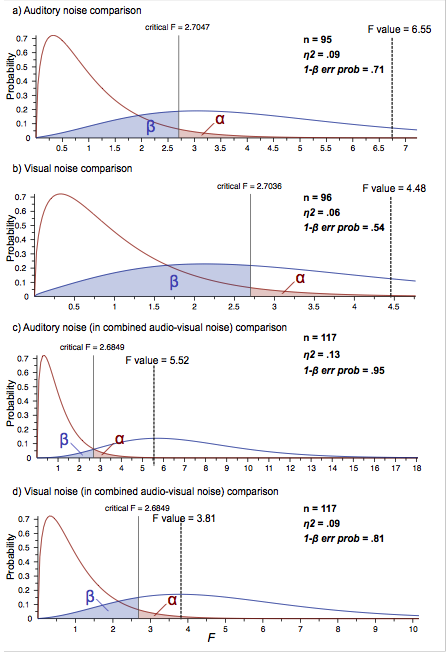


# S6 Exploratory analysis: Are social aptitude and vocabulary knowledge related to the McGurk effect in children

## S6.1 Rationale

The current study enabled secondary, exploratory analyses to investigate whether social aptitude and vocabulary knowledge are associated with McGurk perception in children. Audio-visual integration of speech information has important consequences for language acquisition (i.e. better integration of auditory and visual information may entail better speech recognition). Indeed, speech perception and vocabulary knowledge have been shown to highly correlate in hearing impaired children ^2^. Better speech perception and language ability also likely entails enhanced social ability. In support of this, children with autism spectrum disorder (ASD) ^3,4^, and adults with higher autistic traits show reduced McGurk perception ^5,6^. Based on these findings we predicted that increased McGurk perception, indicating higher audio-visual integration, would correspond to higher vocabulary knowledge and increased social aptitude. Investigating whether these scales are associated with McGurk responses could potentially further our understanding of whether reduced McGurk responses in ASD might be linked to social aptitude or vocabulary skills.

## S6.2 Measures

### Social Aptitude scale (SAS)

The social aptitude scale is an assessment of social ability designed to indicate risk of ASD ^7^. This is a brief 10-item questionnaire completed by parents about their children. Example items include “Able to compromise and flexible” and “Easy to chat which, even if it isn’t on a topic that specially interests him/her”. Parents rate their child from “a lot worse than average”, “a bit worse than average”, “about average”, “a bit better than average” or “a lot better than average” in comparison with other children of the same age. Parents completed this questionnaire either prior to or on the day of data collection.

### British picture vocabulary scale (BPVS)

The BPVS is an assessment of children’s receptive vocabulary knowledge. Children were read aloud a word by a researcher and were asked to point to one of four pictures that they felt represented the word. The researcher started on a set appropriate to the child’s age and then moves through each set until 8 out of 12 items in a set are incorrect. The assessment was conducted with all children in a quiet room at the University in a room with other studies.

## S6.3 Results

### S6.3.1 Do McGurk responses increase with social aptitude?

SAS scores can range from 0 to 40 with lower scores indicating a higher risk of ASD. SAS scores were available for 78 children (M = 24.79, SD=6.25, range 3-40). Correlational analyses were performed to examine whether there was a relationship between SAS scores and fusion (“Da”), visual (“Ga”) and auditory (“Ba”) responses to incongruent McGurk stimuli. Relationships between fusion (“Da”) responses and SAS scores (*r* = .04, *p* = .73), visual (“Ga”) responses and SAS scores (*r* =< .01, *p* = 1) and auditory (“Ba”) responses and SAS scores (*r* = -.05, *p* = .67) were all non-significant.

### S6.3.2 Do McGurk responses increase with vocabulary knowledge?

Raw BPVS scores are thought to increase with age ^8^. To prevent this confound, we used each child’s standardized score, derived using the raw scores and the norms table provided by the BPVS ^9^. Using these scores a score of 100 indicates vocabulary knowledge expected based upon the child’s age, scores lower or higher than this indicate lower or higher vocabulary knowledge than that expected based on age respectively. BPVS data were available for 82 children (M = 104.13, SD=11.85, range 71-134). Correlational analyses were performed to examine whether there was a relationship between BPVS scores and fusion (“Da”), visual (“Ga”) and auditory (“Ba”) responses to incongruent McGurk stimuli. Relationships fusion responses and BPVS scores (*r* = -.154, *p* = .17) and correct auditory responses and BPVS scores (*r* = .194, *p* = .081) were both non-significant. BPVS scores did appear to positively correlate with visual, “Ga” responses (*r* = .227, *p* = .041) – children with higher vocabulary knowledge made more visually driven responses to incongruent McGurk stimuli.

## S6.4 Discussion

We hypothesised a link between audio-visual speech integration, language ability and subsequent social skill, thus, we predicted increased McGurk perception would correspond to higher vocabulary knowledge and increased social aptitude. However, we found neither “Visual”, “Auditory” or “Fusion” responses were correlated with social aptitude score. We also find neither “Fusion” nor “Auditory” responses were correlated with vocabulary knowledge, assessed using the BPVS. There was however a positive correlation between visually driven “Ga” responses on McGurk trials and BPVS score. This preliminary data might be indicative of a link between vocabulary knowledge and visual dominance (participants whose perception was more driven by visual information in the McGurk task also showed higher vocabulary knowledge).

# Supplementary references

1. Barutchu, A. *et al.* Audiovisual integration in noise by children and adults. *J. Exp. Child Psychol.* **105,** 38–50 (2010).

2. Park, H.-J. *et al.* The Relationship between Vocabulary Knowledge and Speech Perception in School-Age Children using Cochlear Implants. *Commun. Sci. Disord.* **21,** 488–501 (2016).

3. Bebko, J. M., Schroeder, J. H. & Weiss, J. A. The McGurk effect in children with autism and asperger syndrome. *Autism Res.* **7,** 50–59 (2014).

4. Williams, J. H. G., Massaro, D. W., Peel, N. J., Bosseler, A. & Suddendorf, T. Visual-auditory integration during speech imitation in autism. *Res. Dev. Disabil.* **25,** 559–575 (2004).

5. Ujiie, Y., Asai, T., Tanaka, A., Asakawa, K. & Wakabayashi, A. Autistic traits predict weaker visual influence in the McGurk effect. *Pers. Individ. Dif.* **60,** S51–S52 (2014).

6. Ujiie, Y., Asai, T. & Wakabayashi, A. The relationship between level of autistic traits and local bias in the context of the McGurk effect. *Front. Psychol.* **6,** (2015).

7. Liddle, E. B., Batty, M. J. & Goodman, R. The social aptitudes scale: An initial validation. *Soc. Psychiatry Psychiatr. Epidemiol.* **44,** 508–513 (2009).

8. Mahon, M. & Crutchley, A. Performance of typically-developing school-age children with English as an additional language on the British Picture Vocabulary Scales II. *Child Lang. Teach. Ther.* **22,** 333–351 (2006).

9. Dunn, L. & Dunn, D. *The British Picture Vocabulary Scale Manual.* (GL Assessment., 2009).

1. University of Nottingham [↑](#footnote-ref-1)
2. Nottingham Trent University [↑](#footnote-ref-2)
3. [↑](#footnote-ref-3)
4. [↑](#footnote-ref-4)
5. CC**orresponding Author Details**

   Miss Rebecca Hirst

   Address:

   School of Psychology

   University of Nottingham

   NG72RD

   Email: Rebecca.Hirst@nottingham.ac.uk [↑](#footnote-ref-5)
6. Greenhouse-Geisser corrected values [↑](#footnote-ref-6)
